# Host-directed therapies in pulmonary tuberculosis: Updates on anti-inflammatory drugs

**DOI:** 10.3389/fmed.2022.970408

**Published:** 2022-09-23

**Authors:** Juan M. Cubillos-Angulo, Betânia M. F. Nogueira, María B. Arriaga, Beatriz Barreto-Duarte, Mariana Araújo-Pereira, Catarina D. Fernandes, Caian L. Vinhaes, Klauss Villalva-Serra, Vanessa M. Nunes, João P. Miguez-Pinto, Eduardo P. Amaral, Bruno B. Andrade

**Affiliations:** ^1^Instituto Gonçalo Moniz, Fundação Oswaldo Cruz, Salvador, Brazil; ^2^Faculdade de Medicina, Universidade Federal da Bahia, Salvador, BA, Brazil; ^3^Multinational Organization Network Sponsoring Translational and Epidemiological Research Initiative, Salvador, Brazil; ^4^Curso de Medicina, Universidade Salvador, Salvador, Brazil; ^5^Programa de Pós-Graduação em Clínica Médica, Universidade Federal do Rio de Janeiro, Rio de Janeiro, Brazil; ^6^Bahiana School of Medicine and Public Health, Bahia Foundation for the Development of Sciences, Salvador, Brazil; ^7^Immunobiology Section, Laboratory of Parasitic Diseases, National Institute of Allergy and Infectious Diseases, National Institutes of Health, Bethesda, MD, United States

**Keywords:** host-directed therapy, *Mycobacterium*, tuberculosis, adjunct therapy, immunotherapies

## Abstract

Tuberculosis (TB) is a lethal disease and remains one of the top ten causes of mortality by an infectious disease worldwide. It can also result in significant morbidity related to persistent inflammation and tissue damage. Pulmonary TB treatment depends on the prolonged use of multiple drugs ranging from 6 months for drug-susceptible TB to 6–20 months in cases of multi-drug resistant disease, with limited patient tolerance resulting from side effects. Treatment success rates remain low and thus represent a barrier to TB control. Adjunct host-directed therapy (HDT) is an emerging strategy in TB treatment that aims to target the host immune response to *Mycobacterium tuberculosis* in addition to antimycobacterial drugs. Combined multi-drug treatment with HDT could potentially result in more effective therapies by shortening treatment duration, improving cure success rates and reducing residual tissue damage. This review explores the rationale and challenges to the development and implementation of HDTs through a succinct report of the medications that have completed or are currently being evaluated in ongoing clinical trials.

## Introduction

Tuberculosis (TB) is caused by infection with *Mycobacterium tuberculosis* (*Mtb*), and represents one of the most important infectious diseases worldwide (1). Until the COVID-19 pandemic, TB was the leading cause of death from a single infectious agent (2).

Throughout the past century, TB morbidity and mortality have declined significantly as a result of a number of factors including improved socioeconomic conditions, introduction of intradermal Bacilli Calmette-Guerin vaccine (BCG), particularly in children younger than 5 years old (3, 4) and most importantly with the introduction of antimycobacterial treatment (5). The use of highly effective therapy against HIV, a co-infection primarily responsible for increased TB incidence and death over the past decades, has also positively impacted TB control (4, 6). Notably, widespread access to anti-TB medications resulted in the closure of inpatient hospitals with a shift to outpatient-based treatment (5).

While worldwide efforts to curb TB incidence and mortality have been effective, the COVID-19 pandemic and subsequent limited access to health services has reversed years of progress in providing essential TB services and reducing TB disease burden (1). In addition, new challenges to control TB include continued insufficient treatment success rates, low treatment adherence and the emergence of drug resistant TB infections (7). In 2020, 132,222 individuals were diagnosed with multidrug resistant or rifampicin resistant TB (MDR/RR-TB) along with 25,681 subjects classified as extensively drug resistant TB (XDR-TB) patient, a decrease in 22% compared with 2019 (201,997) that reflects underdiagnosis of this condition (1).

Current TB treatment relies on a combination of multiple antimicrobial drugs with treatment duration ranging from 6 months for drug-susceptible TB to 6–20 months for MDR/RR-TB and even longer in cases of XDR-TB or poor clinical response (8). Globally, the TB treatment success rate is 85% for drug-susceptible TB and 57% for MDR/RR-TB (1). Outcomes are affected by several factors ranging from social determinants to the long duration and complexity of medication regimens, which directly impact patient adherence to the therapeutic protocol as well as drug toxicity (9). While changing social factors to improve treatment success is a complex, lengthy, and gradual process, development of more effective, affordable, and well-tolerated medications may shorten treatment duration and reduce collateral effects thereby improving treatment outcomes.

Two different strategies have been adopted to develop novel therapeutics: (1) traditional search for new antimycobacterial drugs, (2) host directed therapy (HDT) capable of modulating the immune response to TB as adjuvant therapy to current anti-TB treatment. The development of new anti-TB drugs is a lengthy and costly process (10), thus the study of HDT may offer an effective alternative that is readily available.

**FIGURE 1:**
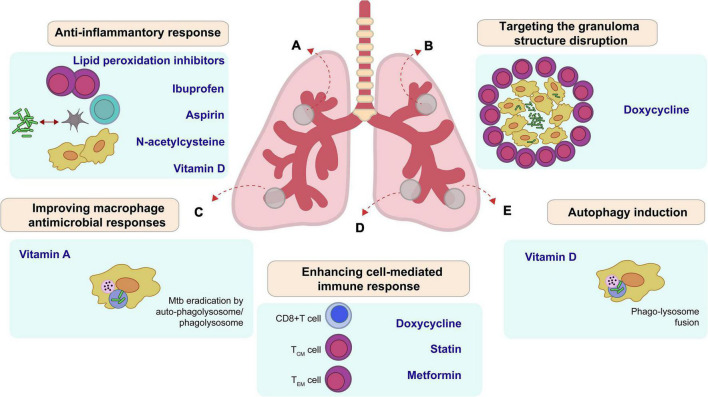
Main potential host therapeutic targets (HDT) improve outcome in *Mycobacterium tuberculosis*. **(A)** Lipid peroxidation inhibitors, Ibuprofen, Aspirin, N-acetylcysteine, and Vitamin D suppress proinflammatory responses, which decrease inflammation and tissue damage during active stage of the disease. **(B)** Doxycycline changes the integrity of granuloma and enhances drug accessibility. **(C)** Vitamin A reduces bacilli growth by apoptosis or auto-phagolysosome. **(D)** Doxycycline, Statin, and Metformin regulate cell-mediated immune responses, including antigen-specific T cell responses. **(E)** Some HDT like vitamin D induce autophagy in infected cells.

Host-directed therapy (HDT) has emerged as an attractive adjuvant treatment option using repurposed approved immune modulation therapy. It has become apparent that the determinants of TB immunopathogenesis and the mechanisms underlying successful infection control involve the following domains: inflammation (Figures 1A–C) (11), cellular metabolism (Figure 1D) (12), and the mechanisms used by *Mtb* to evade the immune system (13; Figure 1E). HDTs aim to modulate host factors to enhance favorable responses and dampen host detrimental responses, which contribute to tissue damage and perpetuation of mycobacterial multiplication (14). If proven to be beneficial, HDT may aid in resolving unmet needs in TB treatment, thereby resulting in improved adherence, reduction of resistant strains, shortened treatment duration and *Mtb* transmission in the community with increased cure rates and fewer chronic sequelae caused by excessive inflammatory response to TB (15).

Currently, there are many potential therapies with ongoing research at different stages in the pipeline for HDT in TB. Most are repurposed drugs in pre-clinical or clinical studies to be used as adjuvants with anti-TB therapy. This review aims to describe the rationale used in the development of HDTs, their potential and main challenges as adjuvant therapy, as well as to provide a succinct report of the medications that have completed or are evaluated in ongoing clinical trials (CT), registered in the ClinicalTrials.gov database.

### Tuberculosis and the immune system

After infection with *Mtb*, the development of active disease results from both pathogen and host factors (16). Immune response against *Mtb* is a very complex and dynamic process, involving different cell types, cytokines and chemokines (17). Multiple inflammatory cells such as macrophages, monocytes, dendritic cells, neutrophils, epithelioid cells, and multinucleated giant cells, enclosed by B and T lymphocytes, accumulate at the tissue level to form a granuloma (18). Myeloid cells produce many cytokines and chemokines that are critical to recruit additional leukocytes from capillary vessels (19, 20). The interaction between differentially localized populations of intracellular *Mtb* and the cellular organelles will dictate whether *Mtb* replicates or restrict its growth through control of intracellular bacilli (21). The majority of *Mtb* exposed individuals contain primary infection with the formation of granulomas. Nevertheless, it is possible that a small proportion of bacilli survive, driving infection into a latent stage. The other potential outcome is increased hypoxic necrotic centers, rich in lipids and foamy macrophages that fail to control bacterial replication, ultimately leading to granuloma caseation (20). This process is responsible for the latter formation of cavities and destruction of alveolar cells, vessels, and bronchi, with consequent bacilli spread (19). The fate of granulomas is determined by a variety of host factors that involve a network of inflammatory cytokines, eicosanoids, prostaglandins, and other mediators contributing to disease exacerbation as well as tissue necrosis (19, 20). Therefore, modulating host immune response could potentially optimize TB treatment, although the ideal target for HDTs remains unclear (22).

Currently, multiple therapies that act on different host immune targets are under investigation including the following: modulation of vascular endothelial growth factor (VEGF) potentially reduces central necrosis and improves drug delivery (23); reduction of neutrophil-mediated inflammation (i.e., aspirin) to limit severe tissue damage (24); and modulation of tumor necrosis factor alpha (TNFα), transforming growth factor beta (TGF-β), and Interleukin-1 beta (IL-1β) may reduce lung damage (25).

### Potential advantages of host-directed therapy in tuberculosis

Some medications studied as HDT in TB are used for other conditions and offer a wealth of clinical experience and research, such as acetylsalicylic acid or statins, to bypass the need to explore the safety and toxicity properties in prolonged use. Furthermore, most of the studied drugs are already available worldwide with accessible costs, facilitating, and accelerating their incorporation into routine practice if benefit in TB treatment is proven in robust CTs (26). The use of HDTs may avoid the undesirable adverse effects with prolonged use of repurposed antimicrobials such as oxazolidonones, carbapenems, and fluorquinolones in TB treatment (27). Among other adverse effects, long-term therapies employing broad-spectrum antibiotics will contribute to the emergence of antibiotic-resistant strains of *Mtb* as well as other opportunistic pathogens (28). Lastly, anti-inflammatory effects offered by some HDT agents may lead to potential benefits in the host by reducing tissue damage and improving long-term quality of life (14).

### The challenge of finding effective host-directed therapy

**FIGURE 2:**
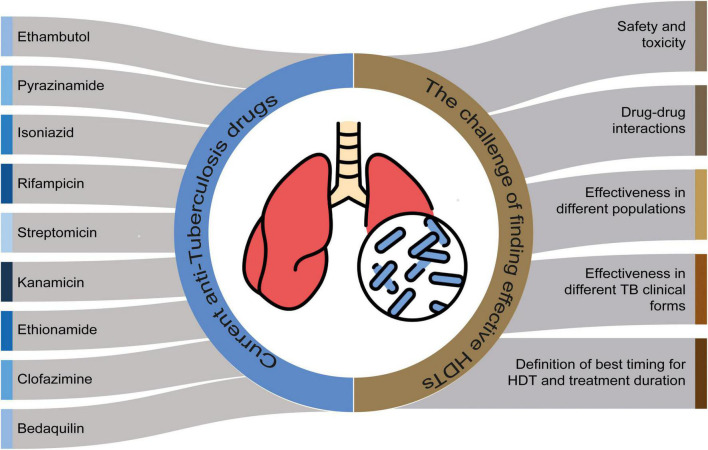
The challenge of finding effective host-directed therapy (HDTs) and the current anti-tuberculosis therapy. Perspectives for clinical studies for HDT efficacy.

*In vitro* studies play an essential role in the screening of potential drugs (29), while animal studies allow the understanding of immunopathology, as well the confirmation of mycobacterial infection control, as measured by the drug impact in mycobacterial load, time to sterilization of lesions, tissue damage size and overall survival (30). With the knowledge obtained through both experimental models, clinical studies to validate these findings will be critical to address questions beyond drug efficacy (31; Figure 2).

#### Safety and toxicity

Though many drugs re-purposed as HDTs have known safety profiles, they must be evaluated in the context of individuals with TB, given that factors such as malnutrition, co-infection with HIV and inflammation tend to alter drug metabolism (32, 33).

#### Drug-drug interactions

The concomitant use of HDT with anti-TB therapy needs to be evaluated. For instance, mild risk of hepatotoxicity of some drugs could be potentiated when used along with anti-mycobacterial drugs. Additionally, it is important to evaluate drug-drug interactions with antiretroviral therapy, as co-infection with TB-HIV is common and of particular concern (26).

#### Effectiveness in different populations

People living with HIV (PWH) and those with other types of immunosuppression, children, and individuals with resistant TB may have different immune responses to TB and thus could respond differently to HDTs (34). Similarly, ethnic differences in TB immune response may also be reflected in different responses to HDTs (35). It is critical that clinical studies include ethnically diverse and clinically relevant populations.

#### Effectiveness in different clinical forms of tuberculosis

While most studies focus on pulmonary TB, extra-pulmonary TB has a high prevalence in some areas, particularly in countries with a high burden of TB (36) with worse outcomes when compared with pulmonary TB (37–39). For instance, having tuberculous meningitis or disseminated TB is associated with lower cure rates and higher mortality rates (37–39).

#### Determine the most effective protocol for host-directed therapy

There may be an ideal time for use during disease course depending on how the host immune response is modulated, and administration in the wrong time frame may be deleterious (31, 40).

#### Evaluation of the possibility of reducing total treatment time

Earlier sterilization and control of inflammation may result in shorter antimycobacterial treatment durations leading to improved patient adherence and increased likelihood of cure (41).

### Drugs targeting the anti-inflammatory response

#### Aspirin

Aspirin is a drug based on acetylsalicylic acid that performs antiplatelet (42), anti-inflammatory (43), and analgesic (44) functions and is a potential adjuvant in the treatment of TB (45, 46).

The anti-inflammatory role of aspirin has been increasingly studied for modulating neutrophil-mediated inflammatory responses. The effect of low-dose aspirin seems to enhance control of bacillary load and improve survival in the late stages of TB in C3HeB/FeJ murine model (45). A study in C3HeB/FeJ mice found that low-dose aspirin had an anti-inflammatory effect in the later stage of active TB by reducing excess, non-productive inflammation, while enhancing Th1-cell responses for the elimination of bacilli (47). In BALB/c mice, aspirin administration enhanced the effect of pyrazinamide and resulted in additional clearance of viable mycobacteria in the lungs and spleen during the initial phase of TB treatment (48). However, the combination of aspirin and Isoniazid-like treatment of murine pulmonary TB was associated with increased mycobacterial load in the spleen and lungs (48). Together, these findings highlight the urgent need for additional clinical studies to assess the impact of timing in disease course and the efficacy of concomitant aspirin use with different anti-TB drug combinations.

In addition to the anti-inflammatory effects, the antiplatelet role of aspirin may also be beneficial in TB treatment given that TB promotes a basal state of hypercoagulability that favors thromboembolic events (49) and platelets have been directly associated with pro-inflammatory status (50). A cohort study of pulmonary TB patients from Taiwan found that low doses of aspirin were associated with decreased morbidity and increased survival of patients on anti-TB regimen, without increasing the risk of bleeding (46). Similarly, a phase two CT in HIV-unexposed adults with tuberculous meningitis found that 1,000 mg of aspirin reduced 3-month mortality rates for this group of patients (51). A hypercoagulation state is present in tuberculous meningitis, leading to vascular complications (49). Using aspirin in this scenario has previously been shown to reduce the incidence of strokes and mortality at 3 months (52).

The results of these preliminary studies are underpowered and require additional robust CTs to prompt a change in current treatment guidelines. Nevertheless, they suggest that aspirin may aid TB treatment in all cases or in subgroups, in those with pulmonary TB or tuberculous meningitis. Importantly, the use of aspirin would be easily implemented given the low cost, high availability and limited side effect profile.

The ClinicalTrials.gov database lists one ongoing multi-center, phase IIB, placebo controlled, randomized CT that aims to evaluate the efficacy and safety of aspirin and ibuprofen as adjunct drugs in TB treatment, as detailed in Table 1.

**TABLE 1:** Clinical trials investigating drugs for host directed therapy in pulmonary tuberculosis (TB).

Adjunctive HDT	Principal investigator and year (last update posted)	Study setting (s)	Trial registration	Type, dose and route of treatment (intervention)	References (PMID or clinical trials website)	Status	Next step drug
Aspirin	(53)	South Africa	NCT04575519	300 mg of Aspirin	https://clinicaltrials.gov/ct2/show/NCT04575519?term=aspirin&cond=Tuberculosis&draw=2&rank=4	Recruiting	Evaluate safety and efficacy of the adjunctive use with TB therapy
Ibuprofen	(53)	Georgia and South Africa	NCT04575519	400°mg Ibuprofen (twice daily during)	https://www.clinicaltrials.gov/ct2/show/NCT04575519?term=ibuprofen&cond=Pulmonary+Tuberculoses&draw=2&rank=1	Recruiting	Determine the impact of ibuprofen on long-term antituberculosis drugs and know the side effects in humans.
N Acetyl Cysteine	(54)	Tanzania	NCT03702738	N-acetylcysteine 1,200 mg	https://clinicaltrials.gov/ct2/show/NCT03702738?term=N+Acetyl+Cysteine&cond=Tuberculosis&draw=2&rank=1	Recruiting	Evaluate the synergize with current therapies in TB and multi-drug-resistant (MDR)-TB treatment
	(55)	Brazil	NCT03281226	N-acetylcysteine 1,200 mg (600°mg twice daily)	https://clinicaltrials.gov/ct2/show/NCT03281226?term=N+Acetyl+Cysteine&cond=Tuberculosis&draw=2&rank=3	Unknown	
Vitamin D	(56)	Indonesia	NCT05073965	1000IU Vitamin D	PMID:35198149	Completed	Need to standardize the doses and optimize the schedule of administration.
	(57)	Pakistan	NCT01130311	Vitamin D (cholecalciferol) 600,000 IU intramuscular	PMID:23331510 PMID:24670704	Completed	
	(58)	Indonesia	NCT00677339	Vitamin D3, “Calciferol Strong^®^” 50,000 IU (1,250 mcg, 1 tablet)	PMID:23967066	Completed	
	(59)	Pakistan	NCT02169570	600,000 IU of (I/M) Vitamin D	https://clinicaltrials.gov/ct2/show/NCT02169570?term=Vitamin+D&cond=Tuberculosis&draw=2&rank=13	Unknown	
	(60)	Mexico	NCT02464683	Vitamin D 200 IU (oral dose)	https://clinicaltrials.gov/ct2/show/NCT02464683?term=Vitamin+D&cond=Tuberculosis&draw=2&rank=2	Unknown	
	(61)	India	NCT00366470	3.3°ml (100,000 IU) dose of Vitamin D	PMID:25863562	Completed	
	(62)	Bangladesh,	NCT01580007	500 mg orally (5,000 IU) Vitamin D	PMID:26394045 PMID:23590701 PMID:29973153	Completed	
	(63)	Ethiopia	NCT01698476	5,000 IU of Vitamin D (cholecalciferol tablets)	PMID:29696707	Completed	
	(64)	India	NCT00507000	Vitamin D 60,000 IU	https://clinicaltrials.gov/ct2/show/NCT00507000?term=Vitamin+D&cond=Tuberculosis&draw=2&rank=5	Unknown	
	(65)	South Africa	NCT02968927	Vitamin D	PMID:33740465	Unknown	
	(66)	Tanzania	NCT00311298	Vitamin D 5 μg/200 IU	PMID:16571156 PMID:22436147	Completed	
	(67)	United Kingdom	NCT03011580	9,600 IU/day Oral Vitamin D	https://clinicaltrials.gov/ct2/show/NCT03011580?term=Vitamin+D&cond=Tuberculosis&draw=2&rank=17	Completed	
Doxycycline	(68)	Singapore	NCT02774993	Doxycycline 100 mg	https://clinicaltrials.gov/ct2/show/NCT02774993	Completed	Results from phase II may provide insights regarding safety and efficacy. New CTs to be performed, including greater sample size and different TB clinical forms besides pulmonary TB.
Vitamin A	(69)	Malawi	NCT00057434	Vitamins A 8,000 IU	PMID:17705950	Completed	Larger CTs looking at effects of Vitamin A in clinical outcomes (death/cure/ relapse)
	(66)	Tanzania	NCT00311298	Vitamin A 5,000 IU	PMID:16571156 PMID:22436147	Completed	
	(70)	India	NCT00801606	Vitamin A 250 mg	PMID:25332327	Completed	
Statin	(31)	South Africa	NCT03882177	Pravastatin 40 mg, 80 mg, 100 mg and 160 mg	https://clinicaltrials.gov/ct2/show/NCT03882177?term=Statin&cond=Tuberculosis%2C+Pulmonary&draw=2&rank=2	Recruiting	Dose finding studies. Phase II CTs are ongoing. If promising results, Phase III trials.
	(71)	South Africa	NCT04147286	Atorvastatin 40°mg	https://clinicaltrials.gov/ct2/show/NCT04147286?term=Statin&cond=Tuberculosis%2C+Pulmonary&draw=2&rank=4	Recruiting	
	(72)	United Kingdom	NCT04721795	Atorvastatin oral 30/40°mg	https://clinicaltrials.gov/ct2/show/NCT04721795?term=Statin&cond=Tuberculosis%2C+Pulmonary&draw=2&rank=1	Recruiting	
	(73)	Philippines, Singapore, Uganda, Vietnam	NCT04504851	Rosuvastatin 10°mg	https://clinicaltrials.gov/ct2/show/NCT04504851?term=Statin&cond=Tuberculosis%2C+Pulmonary&draw=2&rank=3	Not yet recruiting	
Metformin	(74)	Thailand	NCT05215990	Metformin 500 Mg Oral	https://clinicaltrials.gov/ct2/show/NCT05215990?term=Metformin&cond=Tuberculosis%2C+Pulmonary&draw=2&rank=1	Recruiting	Phase II and dose finding studies
	(75)	South Africa	NCT04930744	Metformin hydrochloride 500 mg	https://clinicaltrials.gov/ct2/show/NCT04930744?term=Metformin&cond=Tuberculosis%2C+Pulmonary&draw=2&rank=2	Recruiting	

HDT, host-directed therapy; IU, international unit; NCT, the National Clinical Trial.

#### Ibuprofen

Ibuprofen is a non-steroidal anti-inflammatory drug that inhibits both COX1 and COX2 cyclooxygenases. It is widely used and has an excellent safety profile, even in children (76). In a mouse model mimicking active TB in humans, the use of ibuprofen reduced bacillary load and affected lung area, leading to increased survival (77). These effects are mainly attributed to inhibition of the synthesis of PGE2, which inhibits phagocytosis, bacterial killing, production of nitrite (76) and T-helper 1 cytokines, and production of tumor necrosis factor α (TNF-α) (78). Another study in a murine model noted that ibuprofen enhanced the bactericidal effect of pyrazinamide during TB treatment (48). As noted above, this approach is currently under investigation in a phase II multi-center placebo-controlled trial (Table 1).

#### Antioxidants: N-acetylcysteine and lipid peroxidation inhibitors

N-acetylcysteine (NAC) is a potent antioxidant widely used as a mucolytic agent in chronic obstructive pulmonary disease (COPD) and cystic fibrosis (79). The role of NAC in TB therapy remains under study. To date, prior studies have indicated that NAC might reduce host oxidative responses (80), reduce pro-inflammatory cytokines such as IL-1, IL-6, and TNF-α (81) and have direct antimycobacterial properties (81, 82).

A study conducted in Brazil compared oxidative stress status in plasma of patients with pulmonary TB, latent TB infection, and healthy uninfected individuals (82). Pulmonary TB patients exhibited higher levels of oxidation products and a reduction of antioxidants compared with latent TB cases or uninfected controls. Cultures were exposed to different doses of NAC and the authors found decreased oxidative stress in treated macrophages and reduced mycobacterial growth when exposed to a high concentration of NAC (82). The capacity of NAC to control *Mtb* infection was further tested *in vivo* in a mouse (C57BL/6) model, resulting in a significant reduction of mycobacterial loads in the lungs (82).

In a phase II randomized CT that evaluated the adjuvant use of NAC in hospitalized individuals with TB-HIV (RIPENACTB study) (80), NAC-treated patients exhibited a significant increase in glutathione levels and total antioxidant status along with lowered levels of lipid peroxidation, a toxic process of oxidative stress response. In this study, the adjuvant use of NAC was not unsafe (80). Similarly, another randomized CT in those with newly diagnosed pulmonary TB in India reported a significant increase in glutathione peroxidase levels in patients receiving NAC and conventional anti-TB therapy compared to placebo group (patients under TB therapy only). Patients receiving NAC-based adjunctive therapy exhibited significant reduction of radiological lung infiltration, faster sputum conversion and more regulated immunological response, when compared to the group without NAC. A substantial body weight gain and improved antioxidant status was noted in the intervention group suggesting a potential promising role for NAC as adjuvant anti-TB therapy (83). Moreover, NAC may play a role in preventing hepatotoxicity of anti-TB usual therapy. An Iranian randomized CT (84) evaluated the effect of adjuvant NAC in those undergoing four drug anti-TB therapy compared with those without NAC therapy. Liver enzyme levels including aspartate aminotransferase, alanine aminotransferase and bilirubin were significantly lower following 1 and 2 weeks of NAC treatment. In this study NAC co-administration appeared to reduce the risk of hepatotoxicity commonly associated with anti-TB therapy.

N-acetylcysteine is a low-cost drug that seems to be safe for use in pulmonary TB treatment. Nevertheless, its effectiveness is still to be proven. One CT is currently in progress to clarify this question (Table 1).

### Autophagy induction

#### Vitamin D

Vitamin D (VITD) participates in the reabsorption of calcium from the bone and intestine and has a fundamental role in bone constitution and remodeling (85). It also acts as an immunomodulatory hormone and influences other processes including central nervous system function and cardiovascular health (86, 87).

In the presence of *Mtb*, VITD plays an essential role in activated macrophages and monocytes in response to antigen exposure by enhancing levels of 1,25(OH) 2D in monocyte/macrophages from normal human hosts (88). The increased levels of 1,25(OH) 2D induces the expression of cathelicidin, an antimicrobial protein responsible for killing infectious agents like *Mtb* (89). Some studies have reported that VITD can down-regulate the expression of mTOR protein, thus inducing autophagy (90). Importantly, VITD deficiency has been associated with susceptibility to TB infection in a comparative cross-sectional study that identified a high prevalence of VITD deficiency among newly diagnosed TB patients and in their household contacts (91). To date, 10 completed studies and four ongoing related to VITD and pulmonary TB were identified in the ClinicalTrials.gov records (Table 1).

The use of VITD as adjunctive therapy has had conflicting results regarding improvement of sputum conversion. Faster sputum conversion rates were found in TB patients receiving adjunctive VITD supplementation in a randomized placebo-controlled CT in Indonesia (92) and China (93). Another CT from Bangladesh demonstrated enhanced intracellular killing of *Mtb* in macrophages *ex vivo* in combination with increased sputum culture conversion at week 4 and at week 8 compared to the placebo group (94). This effect, however, was unable to be replicated in a number of CTs of VITD supplementation in TB disease (93, 95–97). Two CTs with MDR/RR-TB patients in Georgia (95) and Mongolia (96) identified higher sputum culture conversion with high dose of VITD use, suggesting a possible role in this subset of patients with MDR/RR TB. Another randomized controlled trial found that daily VITD administration in TB infected patients led to enhanced clinical recovery, particularly those with lower levels of VITD and an elevated TB score at enrollment (97).

Although lower levels of VITD are commonly observed in individuals with pulmonary TB, clinical and bacteriological results from randomized controlled trials of adjunctive VITD supplementation have demonstrated limited clinical benefits. A meta-analysis found that there is no evidence to support the adjuvant use of VITD. Most studies are limited by small sample sizes and include only HIV-uninfected adults with pulmonary TB (98). Additional larger studies are needed to investigate the effects of VITD and other micronutrients in specific TB treatment subgroups that have worse prognosis, immunosupression, MDR/RR-TB and individuals with diabetes.

### Targeting the granuloma structure disruption

#### Doxycycline

Matrix metalloproteinases (MMPs) are proteolytic enzymes capable of degrading collagen and other structural proteins (99). When highly expressed, as in inflammatory conditions, they contribute to tissue damage (100). TB leads to upregulation of MMPs and an imbalance between MMPs and tissue inhibitors of metalloproteinases (TIMPs) (101, 102). This imbalance is associated with TB severity and extent of TB lesions, as well as formation of lung cavitation (103), which in turn is associated with high bacillary burden, delayed sputum culture conversion, emergence of drug resistance, and higher transmission of *Mtb* (104). MMPs also play a role in the formation of granulomas (105) and are responsible for the breakdown of the blood brain barrier, leading to poor outcomes in cases of central nervous system TB (106–108). In this context, inhibitors of MMPs may be a particularly effective HDT for TB. Doxycycline is the only FDA−approved MMP inhibitor (102). It is originally used as a bacteriostatic antibiotic of the tetracycline class. It is also an inhibitor of MMP1 and MMP9 (102) which may be responsible for reducing pulmonary cavity volume and loss of granuloma size (109). Doxycycline has been shown to inhibit mycobacterial growth in animal and *in vitro* models (110, 111). A pilot phase 2 CT (Doxy-TB) (109), comparing doxycycline plus standard TB therapy to placebo plus standard TB therapy has been concluded with results pending (Table 1). Doxycycline is a licensed, safe and affordable drug, with significant potential to improve TB outcomes (112). Future CTs are needed with larger sample sizes and different TB clinical forms besides pulmonary TB, such as central nervous system TB.

### Improving macrophage antimicrobial responses

#### Vitamin A

Vitamin A deficiency is a serious and widespread public health problem (113). It is more common during infection and can increase the severity of infectious diseases and the risk of death (113).

A recently systematic review/meta-analysis found that supplementation with vitamin A associated with earlier sputum conversion, decreased abnormalities in chest radiography, and improved lung function in patients undergoing TB treatment (114). A randomized controlled trial with a 2 × 2 factorial design in Qingdao, China determined that adjunctive supplementation with vitamin A did not improve time to smear conversion in pulmonary TB patients (93). Conversely, a double-blind, placebo-controlled study in patients with newly diagnosed TB found that vitamin A supplementation was associated with earlier sputum smear conversion (115). Furthermore, a randomized placebo-controlled, double-blind, two-by-two factorial trial evaluated the use of multivitamin/mineral supplementation with vitamin A and found a significant decrease in mortality during treatment of sputum-positive TB patients co-infected with HIV (116).

Three CTs registered with ClinicalTrials.gov with the status of completed were identified for Vitamin A supplementation in TB treatment (Table 1). The CT NCT00057434 conducted in Malawi did not identify a survival benefit with micronutrient supplementation with vitamin A in adults with HIV and pulmonary TB (69). Another CT (NCT00311298) found that multi-vitamin/mineral supplementation (Vitamin A) and zinc decrease mortality during treatment in patients with HIV and pulmonary TB (116). The remaining CT (NCT00801606) observed that micronutrient supplementation (Vitamin A) during treatment is associated with weight gain in children with TB though did not impact the clearance of lesions by chest X-ray (117).

These studies highlight conflicting results, though significantly lack evaluation of impact on clinical relevant outcomes (cure/death/recurrence). Larger trials with clinical important outcomes are needed to better evaluate the adjuvant use of vitamin A in TB treatment.

### Enhancing cell-mediated immune response

#### Statins

*Mycobacterium tuberculosis* has been shown to thrive in cholesterol rich environments, as cholesterol can improve both survival and growth of the pathogen in a host cell. *Mtb* uses cholesterol in the macrophage membrane to bind and enter the cell (118). Subsequently, after the infection, macrophage accumulates lipid bodies forming foamy cells that utilize cholesterol as the main source of nutrition for bacteria. The lipid bodies have also been associated with *Mtb* growth restriction, drug resistance and delayed phagosome maturation due to enhanced IL-10 induction (119).

Statins are the most used cholesterol reducing drugs. They are inhibitors of 3-hydroxy-3-methylglutaryl coenzyme reductase (HMG-CoA), with both cholesterol lowering and anti-inflammatory and immunomodulatory functions and offer the most promising candidates for adjuvant HDT against TB (15, 120). The mechanism of action of statins against TB continue to be studied though research to date has identified the following: restrict the generation of foamy cells that otherwise support *Mtb* persistence by decreasing cholesterol biosynthesis (121); promote phagosome maturation and autophagy (122); increase percentages of Natural killer T (NKT) cells within cultures and expression of co-stimulatory molecules on monocytes, with higher secretion of IL-1b and IL-12p70 (123, 124); inhibit TGF-β (125, 126). Animal studies have found that simvastatin therapy in *Mtb*-infected mice reduces dissemination of *Mtb* from the lungs (122), promotes killing of intracellular *Mtb* by macrophages and enhances the bactericidal activity of isoniazid (119) and rifampin (127). A study in mice evaluated the treatment-shortening potential of therapy with statins plus anti-TB drugs and found a reduced time required to eradicate TB infection with no increased risk of relapse (128).

Some retrospective studies in Taiwan and South Korea have evaluated the role of statin use in preventing TB finding that chronic statin users had a lower risk of developing TB, compared to non-statin users (120, 129). Though intriguing, these study findings unfortunately have not been reproduced in different populations distinct from those with COPD and diabetes and lack adjustment for important confounding factors (130–134). A meta-analysis of data from nine of these cohort studies concluded that statin use was associated with reduced incidence of active TB (135). To our knowledge no retrospective studies have evaluated the impact of statins during TB treatment on clinical outcomes.

Four ongoing CTs of statins in TB may offer further clarity (Table 1): StAT-TB, a phase 2B dose finding study using pravastatin (NCT03882177); ATORTUB, using atorvastatin (NCT04721795); ROSETTA, using rosuvastatin (NCT04504851), and StatinTB (NCT04147286), a proof-of-concept phase II study testing the use of atorvastatin in reducing lung inflammation after TB treatment.

Given the pre-clinical studies results and the number of ongoing CTs, statins may represent one of the most promising drugs in the pipeline for HDT. If proven beneficial in clinical studies, its use in clinical practice can be easily implemented, considering its low cost and wide availability though likely will need additional safety testing given the possibility of hepatoxicity.

#### Metformin

Metformin is the first-line therapy in those with type 2 diabetes (136) and is a safe and widely used medication. Results from multiple studies suggest that concurrent use of metformin for TB may be beneficial even in non-diabetic individuals (137).

How metformin acts against TB remains unclear. Pre-clinical studies have found that it facilitates phagosome-lysosome fusion and promotes expression of 5′ adenosine monophosphate-activated protein kinase (AMPK) (14), an enzyme usually activated during metabolic stress that controls energy homeostasis (138). As such, metformin may promote increased production of reactive oxygen species (ROS) and subsequent killing of intracellular *Mtb* (14).

A study performed by Singhal et al. in TB infected mice revealed that metformin reduced intracellular growth of *Mtb*, enhanced the efficacy of anti-TB drugs, and reduced lung damage and inflammation (139). This study served as a proof-of-concept demonstrating that metformin may be an option as adjunctive therapy of TB. Another study evaluating the sterilizing role of metformin found no differences in *Mtb* burden in the metformin adjuvant group versus TB treatment alone (140). Some possible reasons for the divergent results are the different model mouses and the different TB drugs used in both studies. Singhal et al. used a single anti-TB drug (isoniazid or ethambutol), whereas Duta NK et al. employed four drugs, which may have masked a possible role of metformin in sterilization. The inclusion of rifampin may also have altered the pharmacokinetics of metformin (140). Alternatively, it may be that metformin acts in an immunomodulatory role that is more clinically relevant than sterilization. Another study with mice found that metformin enhanced the effectiveness of *Mtb*-specific CD8 + T cell responses in local and systemic sites during infection, with increased decreased mortality and anti-mycobacterial properties and decreased inflammatory cytokine production such as TNF (141). Furthermore, Bohme J. et al. found that metformin enhances the immunogenicity and protective efficacy of BCG in mice (142).

Prior retrospective studies in patients with type 2 diabetes and TB suggest that metformin use was beneficial during TB treatment with reduced risk of cavitary TB (139), more rapid sputum conversion (143), lower mortality despite significantly higher glycated hemoglobin values (139, 144) and lower risk of recurrence (145) when compared to the use of other diabetes treatment regimens during TB treatment.

Considering the evidence above, the drug safety profile, wide availability, and low cost, there remains an important role for CTs to evaluate the effectiveness of metformin as TB adjunctive therapy. Currently, ClinicalTrials.gov reports two CTs investigating Metformin use in individuals with pulmonary TB that are currently recruiting participants in Thailand and South Africa (Table 1).

## Conclusion

Novel TB interventions beyond antimicrobials are urgently needed to improve treatment effectiveness with shorter duration and without increased adverse effects. HDTs offer a promising strategy through repurposing of pre-existing drugs, that often are widely available with low cost, and therefore easily implemented if efficacy and safety are proven in robust CTs. Currently, HDT drugs are in different stages of research with primarily pre-clinical studies with conflicting conclusions. New CTs, ideally multicenter, are underway to answer questions regarding HDT drugs in TB treatment, including all populations of clinical relevance. The existent TB networks or consortia that include standardized cohorts of patients with confirmed TB represent an opportunity for harmonized CTs with heterogenous populations and homogeneous protocols. We suggest the use of these networks or consortia to expedite quality research in this field.

## Author contributions

JC-A, BN, and BB-D contributed to conception and design of the study. JC-A, BN, CF, CV, KV-S, VN, JM-P, MA-P, MA, and EA also collected the information and organized the different subsections. JC-A, BN, and BA wrote the first draft of the manuscript. BA supervised the project execution. All authors contributed to manuscript revision, read, and approved the submitted version.
